# Commentary: Cumulative effects of anodal and priming cathodal tDCS on pegboard test performance and motor cortical excitability

**DOI:** 10.3389/fnhum.2016.00070

**Published:** 2016-03-01

**Authors:** Pierre Besson, Stephane Perrey, Wei-Peng Teo, Makii Muthalib

**Affiliations:** ^1^EuroMov, University of MontpellierMontpellier, France; ^2^School of Exercise and Nutrition Sciences, Deakin UniversityMelbourne, VIC, Australia

**Keywords:** tDCS, motor performance, metaplasticity, neuroergonomics, priming effects

Consistent with a neuroergonomics approach, task performance can be facilitated by non-invasive neuromodulation techniques, such as anodal transcranial direct current stimulation-atDCS (Clark and Parasuraman, 2014; McKendrick et al., 2015). However, robust stimulation parameters and protocols need to be developed for applying atDCS to enhance motor performance in clinical and healthy populations. For instance, protocols using Online atDCS, where the motor task is performed during the stimulation, has greater facilitative effects on motor performance/learning than if the motor task is performed after the stimulation (i.e., Offline atDCS; Stagg and Nitsche, 2011). These greater facilitative effects of Online atDCS on motor performance/learning are likely due to enhanced synaptic efficacy in the simultaneously engaged neural network through a “gating” mechanism (Ziemann and Siebner, 2008). Overall, the interaction of the timing of tDCS application and motor task are crucial parameters to optimize atDCS effects on enhancing motor performance/learning.

The recent study of Christova et al. (2015) aimed to optimize Online atDCS effects on enhancing motor performance/learning by applying a novel cathodal tDCS (ctDCS) priming protocol that harnessed homeostatic metaplastic mechanisms. In the design of the study, healthy subjects were randomly distributed into three priming tDCS groups (*n* = 12) and were required to perform with their non-dominant left hand a grooved pegboard test (GPT) over four training blocks and a retest 2 weeks later. Three priming tDCS conditions were investigated on the right primary motor cortex (M1): (1) Sham: Sham ctDCS (15 min) 10 min before Sham Online atDCS (20 min); (2) Online atDCS: Sham ctDCS (15 min) 10 min before Online atDCS (1mA, 20 min); (3) ctDCS priming: ctDCS (1mA, 15 min) 10 min before Online atDCS (1mA, 20 min). Transcranial magnetic stimulation (TMS) parameters (motor evoked potential-MEP, intracortical facilitation-ICF, and short interval intracortical inhibition-SICI) were assessed before and up to 60 min after the tDCS conditions. The results indicated that although both Online atDCS conditions improved GPT performance (i.e., faster completion time) over Sham after the four training blocks, only the priming ctDCS/Online atDCS condition further enhanced GPT performance 2 weeks later. These latter findings were explained in relation to homeostatic metaplastic mechanisms based on the Bienenstock-Cooper-Munro (BCM) theory that postulates a “sliding threshold” for bidirectional synaptic plasticity (Karabanov et al., 2015). Accordingly, priming with ctDCS, which reduced cortical excitability (reduced MEP amplitude and ICF) and increased cortical inhibition (increased SICI) after the ctDCS session, would have reduced post-synaptic activity in the activated neural network. Based on the BCM model, this ctDCS-induced reduction in post-synaptic activity would be expected to reduce the modification threshold for long term potentiation (LTP)-like plasticity during subsequent Online atDCS, and thus further enhanced GPT performance 2 weeks later. The prolonged increase in ICF and reduced SICI for at least 60 min afterwards provides some evidence for this homeostatic metaplastic effect enhancing offline learning of the GPT. However, the authors acknowledged that a limitation of the study design was that a priming ctDCS followed by Sham Online atDCS condition was not tested, which could have confirmed that the results of the priming ctDCS/Online atDCS condition were primarily due to homeostatic metaplastic mechanisms. Nevertheless, Christova et al.'s (2015) novel methodology and findings can be used to optimize tDCS priming protocols to modulate neuroplasticity and enhance motor performance/learning. The following sections will provide a commentary on ways to optimize the timing and polarity of tDCS applications, which could have significant implications for the original paper's conclusion.

An important tDCS parameter that requires further investigation is the influence of the time delay between priming and test tDCS application on homeostatic metaplasticity and its effects on motor performance/learning (Karabanov et al., 2015). A few studies have investigated the effects of altering the delay between repeated tDCS applications of the same polarity on cortical excitability (Fricke et al., 2011; Monte-Silva et al., 2013; Bastani and Jaberzadeh, 2014) and motor performance/learning (Bastani and Jaberzadeh, 2014). However, no clear evidence of the optimal delay time period could be ascertained from their respective priming tDCS protocols. Christova et al. (2015) considered a 10 min delay between ctDCS and Online atDCS to be sufficient to allow homeostatic metaplastic mechanisms to take hold. But it is still not known if a shorter or longer time delay between priming ctDCS and Online atDCS would differentially modulate homeostatic metaplasticity and motor performance/learning. We (Muthalib et al., 2016) have previously postulated a non-homeostatic approach of priming with atDCS immediately before Online atDCS to further facilitate the neuroplastic effects of Online atDCS. We reason that since sub-threshold neuronal membrane depolarization induced by atDCS has an intensity- and time-dependent effect to strengthen synaptic efficacy (Nitsche and Paulus, 2001), performing atDCS (2 mA, 10 min) immediately before Online atDCS would boost the already strengthened synaptic connections through a further “gating” mechanism induced with the concurrent motor task. We have recently shown that this priming atDCS/Online atDCS protocol on the left M1 can reduce bilateral M1 activation to perform a unilateral simple finger sequence task at the same tapping rate (Muthalib et al., 2016). These results could be explained by a non-homeostatic mechanism following the “gating” theory, such that the reduced motor task related bilateral M1 activation during the atDCS suggests a greater efficiency of neuronal transmission (i.e., less synaptic input for the same neuronal output) in the activated neuronal network. Whether this priming atDCS/Online atDCS protocol would enhance motor performance/learning greater than a priming ctDCS/Online atDCS or Online atDCS protocol still requires to be investigated.

Since the design of the Christova et al. (2015) study corresponded to a learning paradigm, it is difficult to differentiate the tDCS effect from the learning effect on improving online GPT performance during the four training blocks. In order to specifically test the tDCS effect, and minimize the effects of learning, on performance would have been to include a familiarization session to allow the GPT task to become “well learned” and performance stabilize at near maximal levels in all individuals prior to starting the tDCS interventions (Hummel et al., 2010). For highly skilled individuals (e.g., elite athletes, expert operators), it is extremely difficult to improve maximal performance levels since learning has reached relative “ceiling” levels. However, this “ceiling” performance can conceivably be modulated directly using neuromodulation protocols. For example, an excitatory TMS protocol to the dominant left M1, which lead to increased M1 excitability, was able to increase dominant right hand maximal finger tapping rate and reduce the decline of the movement rate over 10 s (Teo et al., 2012). In contrast, an inhibitory TMS protocol to the dominant left M1, which decreased M1 excitability, was shown to decrease maximal finger tapping rate of the dominant right hand (Jäncke et al., 2004). We therefore, consider that applying tDCS to the dominant left M1/right hand and utilizing a “well learned” stable motor task, such as a simple finger sequence task performed at maximum rate (Avanzino et al., 2008), may provide a sensitive means to investigate the tDCS effects on task performance.

In conclusion, priming tDCS protocols are promising ways to optimize tDCS facilitatory effects on motor performance/learning, which has relevance from a neuroergonomic standpoint. Thus, future studies are necessary to determine the optimal polarity and timing of tDCS applications to modulate neuroplasticity and enhance performance in clinical, sports, and real-world settings.

## Author contributions

All authors listed, have made substantial, direct, and intellectual contribution to the work, and approved it for publication.

### Conflict of interest statement

The authors declare that the research was conducted in the absence of any commercial or financial relationships that could be construed as a potential conflict of interest.
